# Sprouts Growing Healthy Habits: Curriculum Development and Pilot Study

**DOI:** 10.3389/fpubh.2019.00065

**Published:** 2019-03-27

**Authors:** Meghan C. Fisher, Elizabeth Villegas, Carolyn Sutter, Salma M. Musaad, Brenda Koester, Barbara H. Fiese

**Affiliations:** ^1^Human Development and Family Studies Department, University of Illinois at Urbana-Champaign, Urbana, IL, United States; ^2^Family Resiliency Center, Human Development and Family Studies Department, University of Illinois at Urbana-Champaign, Urbana, IL, United States

**Keywords:** healthy habits, health education, program implementation, program effectiveness, health curriculum

## Abstract

High rates of childhood obesity and the impact this has on children's health make it important to establish a healthy lifestyle during the early school years. This pilot study explored the impact of a newly developed healthy habits curriculum. The Sprouts: Growing Healthy Habits curriculum presents topics such as energy balance, healthy sleep habits, and food groups through short activities and interactive book-reading. A nonrandomized controlled experimental study design was used to assess impact. Fifty-seven children between 5 and 6 years of age participated from two elementary schools (36 from the intervention school, 21 from the control school). Knowledge was assessed pre- and post-intervention in five content areas (farm to table, bedtime routines, portion size, energy expenditure, sugar content of beverages) using card sorting, ranking, and sequence activities. Within- and between-school comparisons were conducted using differences between groups and mixed modeling approaches. Within the intervention school, significant increases in knowledge were observed for farm to table, sugar content of beverages, and bedtime routines. For the control school, there was a significant increase in knowledge of portion sizes. Considering between-schools, only change in knowledge of bedtime routines remained significant, with greater increases in the intervention school. Results seem promising given the short dosage of curriculum. Increases in knowledge of healthy habits in early childhood may help in promoting healthy behaviors and combatting the effects of obesity.

## Introduction

Childhood obesity continues to be a major issue in the United States especially during early childhood with approximately 18.4% of 2–5 year olds and 20.6% of 6–11 year olds meeting criteria to be considered obese (1). Ninety-five percent of the nation's children are of school age, with 4.2 million children in kindergarten classrooms, suggesting schools are a prime setting to assist in addressing today's obesity issue among children early on (2). During the early childhood years children learn about food, establish health behaviors, and develop food preferences that can track into adulthood (3, 4), thus making this an ideal time period for obesity prevention efforts. Additionally, children who are overweight in childhood are at a significantly higher risk of being overweight or becoming obese in adulthood (5). Therefore, there is a need to address practices that help children learn about nutrition and establish healthy habits early on to impact their dietary and behavior trajectories later in life. Three primary factors related to childhood obesity are dietary patterns, involvement in food preparation, and behavioral patterns including energy balance and sleep routines, which will be discussed in further detail.

The overweight status of children has been linked to lifestyle habits including unhealthy dietary patterns. Diets consisting of energy-dense, sugary, low-quality foods (e.g., salty snacks like potato chips, sweets, fats/oils), and dietary patterns with an increased total amount of food (i.e., large portions, more snacks) are associated with children being overweight (6). Not only is increased food consumption a concern, but consumption of sugar-sweetened beverages (SSBs) needs to be considered when discussing factors affecting childhood obesity. SSBs have been shown to have a positive relationship with both children's body mass index (BMI) and their weight (7, 8). Sugar-sweetened carbonated beverages have also been linked to less exercise in boys and poorer diet choices in both girls and boys (9). Studies suggest that nutrition education and food knowledge are associated with actual diet, with children as early as 4 years of age starting to understand the difference between healthy and unhealthy food, such as choosing fruit verses crackers for a snack (10, 11). This indicates a need for educators and parents to teach children about these topics, such as portion sizes and high energy-dense verses high nutrient-dense foods and beverages, to increase knowledge and encourage healthy dietary patterns.

Teaching children about healthy choices for their diet and routines can impact childhood obesity as well as long-term health. The knowledge that promotes healthy choices is not limited specifically to nutrition but also includes teaching children about where foods come from and how to prepare food. Children who participate in garden programs have shown an increase in the willingness to try new foods, especially if they grew it or cooked it themselves (12). Morris et al. (13) found that school-based gardens can lead to healthier eating habits; children who were involved in school gardens showed improved preference of vegetables that lasted 6 months after involvement. Having the knowledge to prepare and cook food is also a valuable skill to possess and could affect dietary patterns. If a person has insufficient skills to prepare food, this could limit types of food consumed, and thus could result in a poor-quality diet (14). Research has shown that preschool-aged children who are actively involved in grocery shopping and simple food preparation are more likely to eat fresh fruits and vegetables (15). Hands-on cooking activities can be a successful way to improve eating habits in youth (16). Larson et al. (17) suggest that adolescents may benefit from programs that teach cooking skills and how to make healthful food purchasing decisions. They found that adolescents who prepared food at home had lower intakes of fat and higher consumption of fruits and vegetables (17). Middle-school youth who participated in a Cooking Camp program demonstrated increased confidence and mastery in food skills, vegetable consumption, and enjoyment in preparing food (18). Providing children with the ability to understand where food comes from, as well as teaching them skills to prepare their food early on, could be beneficial in making healthy dietary choices throughout life.

Establishing a healthy lifestyle does not only refer to following appropriate dietary patterns, but also includes energy balance. Energy balance is defined as the balance between energy intake and expenditure, which plays an important role in maintaining a healthy lifestyle. Time allocated to both physical activity and screen time can influence the expenditure aspect of energy balance. Janssen et al. (19) showed that decreased amounts of physical activity and increased amounts of television viewing were associated with a higher likelihood of being overweight, even when controlling for dietary patterns. Television viewing among children is at an all-time high with children in front of a screen approximately 4.5–7 hours a day (20). The amount of time children spend looking at a screen, including televisions, computers, and other electronic devices, has increased dramatically and taken over children's free play time, once used for physically active play. Additionally, watching television during mealtimes is shown to be related to dietary patterns of children, specifically an increased amount of salty snacks and soda intake, and decreased amount of fruits and vegetables being consumed (21). The numerous studies that have found links between childhood obesity and increased television viewing, coupled with the alarming amount of hours children spend in front of a screen, further supports a need to address screentime patterns with children (22, 23). Energy balance-related behaviors are contributors to the childhood obesity epidemic, and thus are important to include in prevention programs.

Research has shown that not only do dietary patterns and amounts of physical activity impact the likelihood of children becoming overweight or obese, but there is also an association with sleep patterns and childhood obesity. Based on an analysis of optimal sleep duration cut-points, a review of 17 studies showed children who had less than the analysis' recommended amounts of sleep (for children under 5 years greater than or equal to 11 hours) had a 58% higher risk for being overweight or obese (24). In addition, children who slept less or had poor sleep continuity were more likely to increase their food and SSBs consumption (9, 25). Sekine et al. (26) found that a decreased amount of sleep in children was related to obesity even when taking into account physical activity and television watching, justifying the need to promote healthy sleep habits for children.

Numerous factors play a role in the childhood obesity epidemic, and to address just one in a prevention strategy would not do the effort justice. In an attempt to overcome the lack of obesity intervention programs focusing on multiple factors during early childhood, the Sprouts: Growing Healthy Habits curriculum was developed by researchers at the University of Illinois at Urbana-Champaign to provide a holistic approach to adopting a healthy lifestyle for children, with an emphasis on incorporating not only dietary patterns (i.e., food and drink consumption, correct portion sizes, food groups), but also healthy behaviors (i.e., physical activity, energy balance, healthy screentime, healthy sleep time routines) into the program components. The goal of this study is to explore the Sprouts curriculum's impact on children's knowledge in healthy habit areas.

## Materials and Methods

### Study Design

The current study being reported is the controlled intervention examining changes in knowledge related to the Sprouts curriculum. Prior to the controlled intervention phase, lessons and assessment tools were piloted and informally tested for feasibility. Teachers who reviewed the lessons reported that the lessons would be age-appropriate, meet educational goals, and fit into existing curriculum (27). Additionally, pilot testing of the lessons and assessments indicated they were feasible for use in kindergarten classrooms and held children's attention. After modifying the curriculum lessons and assessment tools based on feedback, the current pilot study was conducted. This study employed a nonrandomized controlled experimental design and was conducted during the 2014–2015 school year in six kindergarten classrooms of two elementary schools.

Prior to curriculum implementation, undergraduate students enrolled in a two-semester transdisciplinary applied research course were trained by project coordinators on either the curriculum intervention or the assessment measures. Different students were assigned to assessing the children and facilitating the curriculum to avoid any leading or biases during the evaluation process. Students who taught the curriculum were from a variety of disciplines at the University of Illinois. Classroom teachers were present during the curriculum implementation but not involved in teaching the lessons. They were asked to not make any changes to their planned curriculum nor to reinforce topics covered in the Sprouts curriculum facilitation phase. We wanted to try to make sure lesson topics were not discussed any more than they normally would, to minimize differences between the intervention and control schools. Classroom teachers were asked to fill out a brief survey after each lesson to provide feedback. Additionally, a student was assigned to complete a curriculum facilitation checklist during every lesson to ensure fidelity of program implementation. These checklists confirmed that all lessons were consistently delivered based on instructions provided. The control school did not receive any type of alternative education in place of Sprouts curriculum. The control group only participated in the pre- and post- assessments.

Signed informed consent was obtained from the participants' parents; assent was provided verbally by children before each assessment. Consent letters were sent home with the kindergarten students at the beginning of the school-year, and parents mailed them back and included their home address to send payment and future surveys. Parents were able to receive $45 in compensation, in the form of gift cards for participating in the project ($15 for initial consent and $15 per completed survey, one sent during each of the two semesters). Prior to data collection, the study was approved by the Institutional Review Board at the University of Illinois at Urbana-Champaign.

Pre and post-tests were exactly the same and consisted of six different assessments measuring healthy habit knowledge associated with the Sprouts lessons. Assessments were conducted across two testing periods with each child due to the amount of time it took to complete all assessments.

### Curriculum Component

The Sprouts curriculum was taught in three kindergarten classrooms in a small metropolitan public STEM academy, during normal school hours by undergraduate students from the University of Illinois. STEM schools focus on educating students in four specific areas: science, technology, engineering, and mathematics, through an interdisciplinary and applied approach. The curriculum consists of eight lessons (see Table 1), taught weekly to individual classrooms for approximately 45–60 min, focusing on children's daily routines to instill the fundamental knowledge of healthy habits in various components of everyday life. The goals of the curriculum are to increase students' knowledge on topic areas, with the long-term goal of impacting behavioral change and reducing rates of childhood obesity. Topic areas include positive family interactions centered around mealtimes, healthy eating habits, understanding where food comes from, managing energy balance, healthy screen time and healthy sleep routines. Each lesson has a specific focus that incorporates a discussion, activity, and a book to keep children engaged while offering a comprehensive approach to teaching healthy habits to this age group. For example, during the discussion section of the lesson “Food Groups and Shopping,” the teacher would ask children about their experiences picking up and choosing foods at a food store and more ways they could be involved in this process. The teacher then teaches the children about the five food groups, the importance of each group, and food examples for each group. During the activity portion of the lesson, children have the opportunity to go down pretend food aisles at a mock store to choose food (paper pictures) from each of the five food groups to make their own meal. The teacher then reads a selected book to the children as a group, and asks them questions from the book related to the lesson. Materials and a lesson outline, that includes discussion questions are included in the curriculum for each lesson. The lesson is concluded by a short recap. All lessons follow this same format. Newsletters were also sent home weekly to inform parents of the healthy habits that their children were learning in school. These newsletters provided parents with strategies on how to integrate healthy habits at a family level.

**Table 1:** Sprouts: Growing Healthy Habits *Lesson and objective overview*.

**Lesson**	**Objective**	**Activity**
Farm to table	Children will learn where our food comes from, understanding each step from farming to ending up on one's table	Activities include an interactive crafting layout and planting radish seeds
Food groups and shopping	Children will understand what types of food fall under each food group and will learn how to make a balanced meal	Activities include a shopping experience in which children get to go shopping in play store to make a balanced meal
Family mealtimes	Children will talk about the importance of shared mealtimes and learn how they can help with food preparation	Children get to participate in making an easy recipe and sharing in a family-style meal conversation
Picky eating	Children will learn about different types of fruits and vegetables and the importance of trying new things for health	Children will get to try a rainbow of fruit options to experience what a variety of fruit tastes like
Snacks and beverages	Children will learn the difference between snacks and meals and explore the differences in sugar content in a variety of beverages	Activities include a guessing game where children separate sugar cubes representing added sugar in each drink
Energy balance	Children will be able to determine which activities use more energy and how food intake needs to be balanced by energy expenditure	Children will get to draw and color foods consumed and favorite activities for energy expenditure
Healthy screentime habits	Children will comprehend what screentime is and how screen time is related to health outcomes	Children will brainstorm ideas of other activities to do besides screentime
Healthy sleep habits	Children will learn about healthy bedtime routines and the importance of these routines	Children will get to have fun with crafts and fill out a worksheet based on bedtime habits

### Participants

Children (69 total) in the intervention group from three kindergarten classrooms were taught the Sprouts curriculum, and had the opportunity to participate in the evaluation piece. However, only the children who assented and had parent consent participated in the pre- and post-intervention assessments (36 children). The low response rate was likely due to the passive recruitment tactics used (i.e., sending consent form home with child in his/her backpack). Children from the control school (*n* = 21) were recruited in a similar fashion but were not given the Sprouts curriculum. Thus, 57 children were included in the analyses. Child demographic information was linked to a parent survey sent out twice (pre-post intervention), thus for any parent who did not send back the survey, there is missing demographic information for their children (approximately 12% of the children). Children engaged in evaluation activities individually with researchers in a separate room from the classroom. Researchers administering the evaluation pieces were trained undergraduate students who were trained and observed for implementation fidelity by a research coordinator overseeing the project. They were not part of the curriculum implementation. During training, the undergraduate students had the opportunity to practice implementing the activities either in groups or with children at a local early childhood school prior to working with the children who participated in this project.

### Measures

All measures were created to assess knowledge change in curriculum topic areas. Items involved in the assessments, such as pictures of specific foods, were the exact pictures that were shown during the curriculum implementation. This was done to prevent any issues with children not being able to recognize items, such as certain foods or activities, based on differences in backgrounds or access to varieties of foods and activities at home.

#### Farm to Table Knowledge

To assess children's knowledge of where food comes from, children were provided with six steps corresponding to the journey a seed takes to become food that is ready to eat at your table and asked to use sequence cards to put these steps in order. The sequence cards were (1) Plant, (2) Grow, (3) Harvest, (4) Deliver, (5) Shop, (6) Eat. Each card had a picture of the action and was labeled with the word. The measure required a child to place the sequence cards in order from first step to the sixth step. For each sequence assessment, research staff laid out the six steps and then identified what each card (step) was, by reading the label on the card, before the children began. The children were asked to place the cards on a board that had designated squares labeled 1 through 6 to assess their knowledge on the process of how food reaches one's table. Each of the six steps was scored on the distance between the child's answer and the correct answer, and then the absolute value was calculated for each item's score. For example, if a child put the second step as the first step, the child would receive a score of one (i.e., 2–1 = 1, and |1| = 1) for the first item. For example, if a child put the third step as the fourth step, the child would receive a score of one also (i.e., 3–4 = −1, and | −1 | = 1). The six-item scores were then summed, with a lower score indicating the child performed better, and a score of zero indicating the child identified each step correctly. The possibility of scores ranged from 0 to 18. A similar sequence assessment scoring was used for the SSBs and energy expenditure assessments described below.

#### Content of Sugar-Sweetened Beverage Knowledge

Knowledge of SSBs was also assessed through a card sequence task. Children were provided with six cards of different types of beverages. The cards were (1) Water, (2) White Milk, (3) 100% Juice, (4) Chocolate Milk, (5) 10% Juice Box, (6) Soda Pop. Each card had a picture of the beverage and was labeled with the type of beverage. The measure required a child to place the cards in order from the least amount of added sugar to the most amount of added sugar in a beverage. This assessment was conducted and scored based on the previously described sequence assessment (see Farm to Table Knowledge description).

#### Portion Size Knowledge

Children were provided with 10 cards, given one at a time. Each card had a picture of either a snack or meal portion size of food. The measure required a child to indicate whether the picture was one of a snack or meal. Research staff identified what each card was, by identifying the items on the picture. For example, a snack card would include items such as a banana, granola, fruit, and cheese, while a meal card would include items such as spaghetti and meatballs with a side plate of salad or eggs, toast, bacon, and fruit. The 10 items were scored with 0 (incorrect) or 1 (correct). The 10 items were then summed and higher scores indicated that the child performed better (Cronbachs alpha pre-test: 0.41, post-test: 0.41). The possibility of scores ranged from 0 to 10.

#### Energy Expenditure Knowledge

Knowledge of energy balance was assessed by providing children with six cards of different types of activities. The cards were (1) Watching TV, (2) Making a craft, (3) Playing with blocks, (4) Walking, (5) Swimming, (6) Playing Soccer. The measure required a child to place the cards in order from the least amount of energy expenditure to the most amount of energy expenditure. This assessment was conducted and scored based on the previously described sequence assessment (see Farm to Table Knowledge description).

#### Bedtime Routine Knowledge

To assess knowledge of bedtime routines, children were provided with 17 cards, given one at a time. Each card had a picture of an activity that is commonly done before bedtime (e.g., have a snack, watch TV, turn off the lights, brush teeth). The measure required a child to indicate whether the picture was something that would help people get a good night's sleep or not. Research staff identified what each card was, by identifying the items on the picture. For example, a card would have a picture of a kid getting tucked in, and the researcher would ask “do you think getting tucked in would help this person get a good night's rest?” The child was then prompted to respond with a yes or no. There were a total of 9 healthy routine cards, that would help a person get a good night's sleep and a total of 7 unhealthy routine cards that have been found to impair quality sleep. The 16 items were scored with 0 (incorrect) or 1 (correct). The 16 items were then summed and higher scores indicated that the child performed better (Cronbach's alpha pre-test: 0.68, post-test: 0.82). The possibility of scores ranged from 0 to 16.

## Analysis

Change in knowledge was assessed as post- minus pre-assessment score for each healthy habits content area. For the portion size and bedtime routine knowledge change scores, higher numbers indicated increases in knowledge. Since knowledge scores for the farm to table, energy expenditure, and SSBs were coded such that values closer to zero indicate the child performed better, negative values for the post minus pre change score indicated increases in knowledge. To ensure that all change scores could be evaluated in the same way, these change scores were recoded as the inverse value such that higher scores also indicated increases in knowledge. Shapiro-Wilk tests indicated that the outcome variables were not normally distributed, and as such non-parametric tests were examined. Differences between pre- and post-intervention scores were examined separately for the intervention and control school using the Signed Rank test (Mann-Whiney U-test). Wilcoxon Two-Sample tests were used to examine change in assessment scores between the schools.

## Results

Differences in demographic characteristics of participants were examined between the intervention and control schools using Fischer's Exact Tests (see Table 2). Participants from the two schools did not differ in terms of child gender, race, or Hispanic/Latino ethnicity. Significant differences were found between participants in the two schools in relation to parent education and household income. Parents in the intervention school reported higher education levels (44.4% college graduates or postgraduate work, vs. 14.3% in the control school) and household incomes (30.6% reported incomes of $100,000 or more, vs. 4.8% in the control school).

**Table 2:** Demographic information compared between control and intervention schools using Fisher's exact test.

	**Control school (*****n*** **=** **21)**		**Intervention school (*****n*** **=** **36)**		
	***n***	**% of sample**	***n***	**% of sample**	***p*-value**
**Child gender**					0.29
Female	8	38.1%	13	36.1%	
Male	8	38.1%	20	55.6%	
**Child race**					0.12
American Indian or Alaskan Native	0	0%	1	2.8%	
Asian	0	0%	4	7%	
Black or African American	8	38.1%	14	38.9%	
White	8	38.1%	14	38.9%	
**Hispanic/latino**					0.14
Yes	2	9.5%	0	0%	
No	15	71.4%	33	91.7%	
**Parent education**					0.03
High school or less	8	38.1%	5	13.9%	
Some college	6	28.6%	12	33.3%	
College grad/ postgraduate	3	14.3%	16	44.4%	
**Household income**					0.01
Prefer not to answer	0	0%	6	16.7%	
$24,999 or less	6	28.6%	11	30.6%	
$25–$69,999	8	47.1%	4	12.1%	
$70,000 or more	3	17.6%	12	36.4%	

### Intra-school Comparison

Table 3 shows the difference in the assessment scores within-schools. Differences from pre- to post-intervention in the intervention school for farm to table, SSBs, and bedtime routine knowledge were significant and in the expected direction. On average, farm to table knowledge scores and knowledge of sugar content of beverages increased by about 2.5 points, and bedtime routines knowledge increased by close to two points. Change in portion size knowledge was also significant within the control school, with on average 1 point increases in knowledge.

**Table 3:** Change in child knowledge in control and intervention schools compared within-school using signed rank tests and between-school using wilcoxon two-sample test.

	**Control school**			**Intervention school**			**Between schools**
	**Mean**	**SD**	***p*-value**	**Mean**	**SD**	***p*-value**	***p*-value**
**Farm to table**							
Pre	5.81	6.10		4.56	4.55		
Post	3.43	4.30		2.11	3.07		
Change	2.38	5.78	0.05	2.57	3.87	< 0.001	0.79
**Bedtime routines**							
Pre	11.57	3.11		12.33	2.14		
Post	11.81	3.37		14.22	4.02		
Change	0.24	1.97	0.49	1.89	4.29	0.002	0.004
**Portion size**							
Pre	7.29	1.52		8.03	1.76		
Post	8.29	1.49		8.17	2.10		
Change	1.00	1.52	0.01	0.14	2.39	0.24	0.10
**Energy expenditure**							
Pre	9.24	3.97		8.33	5.16		
Post	8.86	5.12		7.49	4.91		
Change	0.38	5.50	0.67	1.09	5.03	0.28	0.86
**Sugar content of beverages**							
Pre	9.62	4.54		7.67	4.82		
Post	8.00	4.77		5.26	4.00		
Change	1.62	5.64	0.18	2.51	5.21	0.01	0.97

### Inter-school Comparison

When considering changes in knowledge from pre to post between the intervention and control schools, there was only a significant difference for bedtime routines knowledge (see Table 3). On average, children who participated in the Sprouts curriculum increased their knowledge of bedtime routines by close to 2 points (*M* = 1.89, *SD* = 4.29) moving from a mean of 12 to 14 points (possible range = 0–16) as compared to children in the control school.

Differences in changes in knowledge from pre- to post-intervention between the intervention and control schools were also examined using separate Generalized Linear Mixed Models for each outcome in order to adjust for household income and parental education. Results and statistical inference from these models were consistent with the Wilcoxon two-sample tests; the only significant change was observed for bedtime routine knowledge, which was higher in the intervention school by about 15%. As such the Wilcoxon two-sample inter-school comparison models are presented for the sake of parsimony and ease of interpretation.

## Discussion

This evaluation provides promising results for the impact of the Sprouts curriculum in kindergarten classrooms. The curriculum was significantly associated with increased knowledge of farm to table, SSBs, and bedtime routines in the intervention school. Unexpectedly, a significant increase in knowledge related to portion sizes and a marginally significant (*p* = 0.05) increase in knowledge of farm to table were also observed in the control school. Activities and lessons in the control school were not monitored during the study period, so it may be the case that children in this school learned about these topics. The Sprouts curriculum related to portion sizes is currently being reexamined and updated to focus more specifically on knowledge of snacks vs. meals and portion sizes, given connections between portion size and overall dietary intake (28).

Furthermore, when looking at the change in outcome across intervention and control schools within the same model, the increase in knowledge remained significant only for bedtime routines. It may be that topics children experience every day, such as bedtime routines, are easy to absorb and remember while newer topics (such as farm to table) that children may be learning for this first time could require repeated exposure in order to see significant increases in knowledge. Sprouts: Growing Healthy Habits can provide a basis for introducing healthy habit topics to children, and future research will need to examine whether multiple lessons can increase knowledge over time. This curriculum can be a helpful tool in teaching children healthy habits that have not been traditionally taught in schools. Furthermore, this curriculum, which implemented lessons once a week that took less than an hour, still made an impact on knowledge, suggesting that with continued reinforcement and emphasis in the classroom and at home, children's knowledge could have continual improvements.

### Study Strengths and Limitations

Lack of observed increases in knowledge in all measures could be attributed to factors other than the effectiveness of the curriculum. For example, in the current study, significant demographic differences were noted between the intervention and control school, making it difficult to determine if increases in knowledge are generalizable to other contexts. However, findings from the mixed models confirmed that, compared to the control school, scores in bedtime routine knowledge increased in the intervention school by about 15%, after adjusting for parental education and household income. This finding strongly suggests that the increases in bedtime routine knowledge are robust, but further research is needed with demographically diverse schools to determine whether the curriculum would increase knowledge for other concepts. Additionally, each site only had a small portion of families consent to the evaluation piece, resulting in a small, demographically limited sample size. As such, it is unknown how the results reported here might generalize to other populations with different demographics. Replication on a larger scale is suggested to provide stronger support for implementing Sprouts to teach about topics including both risk and protective factors related to obesity, in hopes that children adopt a healthy lifestyle.

It should also be noted that the assessment tools used to examine changes in knowledge related to curriculum implementation were developed specifically for the current study. This is both a strength and a limitation. Given that no measures appropriate to capture change in child knowledge in the healthy habit domains covered by the Sprouts curriculum were available at the time of measurement, this study provides a first step toward creating measurement tools for use in early childhood settings to evaluate changes in children's knowledge of healthy habits. However, the current pilot study was limited in scope in terms of the ability to carry out extensive reliability and validity testing of the developed assessment tools. More work is needed to develop valid measurement tools in these areas. As such, changes in knowledge (or lack thereof) observed with these assessment tools, such as the portion size measure which had low internal consistency, should be interpreted with caution.

Another component that may impact measurement is the aptitude level of the child. We were unable to measure children's language comprehension and cognitive ability in order test whether the children fully comprehended the lesson topics or the measurement tools. Furthermore, the intervention would be best optimized if classroom teachers and parents reinforced health messages throughout the week or time period outside of when curriculum was being taught. Studies have shown that children learn best when material has been repeated and taught in multiple formats (i.e., activities, stories, songs, discussion), therefore it would be helpful to understand how often a topic needs to be reinforced to make a difference for a child's health knowledge and behavior (29).

Due to the many contributing factors to childhood obesity, there are many topic areas that need to be covered in a healthy habits curriculum. The curriculum's eight lessons are each focused on a different topic area that is part of what constitutes a healthy lifestyle. We measured knowledge change shortly after the entire curriculum was taught, and thus, we do not have a measure of sustained results over time. There were significant changes seen from pre to post, even though there was limited dosage of each topic lesson, showing positive results at least for short-term knowledge gain. In future research, studies should measure behavior change in parallel with knowledge change, as in the current study the main objective was to first understand knowledge change. Long-term follow-up is also necessary to build a better understanding of whether school-based curriculum is the best avenue to create sustainable interventions to make lasting knowledge and behavior changes for children and their families. It may be necessary to also target parents through outreach materials or involve parents in classroom lessons in order to establish healthy habits that extend across contexts. Despite these limitations the results show promise for this curriculum.

Considering that, the Sprouts program showed promise in increasing knowledge the next steps are to adapt the curriculum for broader audiences. We recognize that a properly tailored curriculum in other early childhood education settings, such as home-based or center-based childcare facilities require adaptation. Therefore, it is necessary to expand on these limitations by connecting with childcare providers and teachers about the barriers and facilitators of incorporating and implementing this curriculum to their specific setting. Adapting the curriculum to a specific setting, demographic, or cultural environment can provide a more open learning environment. Additionally, beyond understanding students' change in knowledge, future studies and educational settings should consider how this curriculum contributes to behavioral change, although this may call for the participation of parent and caregivers. Including these elements could provide the evidence needed to promote healthy habits curriculum in early childhood education nationwide.

## Conclusion

We believe that one of the best ways to combat childhood obesity is through prevention at an early age, given that changing ingrained lifestyle habits at a later age has been shown to be extremely difficult. This curriculum shows promising results with young children in a short amount of time. The Sprouts: Growing Healthy Habits curriculum covers a variety of topics related to healthy habits that has shown the ability to increase knowledge in these areas. This curriculum may be useful to teachers looking for a way to teach young children about a range of health topics that promote a healthy lifestyle.

## Data Availability

The datasets generated for this study are available on request to the corresponding author.

## Author Contributions

MF, BF, and BK contributed to the conception and design of this study. MF and EV managed the data collection of this study. EV organized the data. MF wrote the first draft of the manuscript. EV, CS, and SM wrote sections of the manuscript. CS and SM conducted the data analyses. All authors contributed to the manuscript revisions, and approved the submitted version.

### Conflict of Interest Statement

The authors declare that the research was conducted in the absence of any commercial or financial relationships that could be construed as a potential conflict of interest.
